# Structural basis of a dual-function type II-B CRISPR–Cas9

**DOI:** 10.1093/nar/gkaf585

**Published:** 2025-07-04

**Authors:** Grace N Hibshman, David W Taylor

**Affiliations:** Department of Molecular Biosciences, University of Texas at Austin, Austin, TX 78712, United States; Interdisciplinary Life Sciences Graduate Programs, Austin, TX 78712, United States; Department of Molecular Biosciences, University of Texas at Austin, Austin, TX 78712, United States; Interdisciplinary Life Sciences Graduate Programs, Austin, TX 78712, United States; Center for Systems and Synthetic Biology, University of Texas at Austin, Austin, TX 78712, United States; LIVESTRONG Cancer Institutes, Dell Medical School, Austin, TX 78712, United States

## Abstract

Cas9 from *Streptococcus pyogenes* (SpCas9) revolutionized genome editing by enabling programmable DNA cleavage guided by an RNA. However, SpCas9 tolerates mismatches in the DNA–RNA duplex, which can lead to deleterious off-target editing. Here, we reveal that Cas9 from *Francisella novicida* (FnCas9) possesses a unique structural feature—the REC3 clamp—that underlies its intrinsic high-fidelity DNA targeting. Through kinetic and structural analyses, we show that the REC3 clamp forms critical contacts with the PAM-distal region of the R-loop, thereby imposing a novel checkpoint during enzyme activation. Notably, *F. novicida* encodes a noncanonical small CRISPR-associated RNA (scaRNA) that enables FnCas9 to repress an endogenous bacterial lipoprotein gene, subverting host immune detection. Structures of FnCas9 with scaRNA illustrate how partial R-loop complementarity hinders REC3 clamp docking and prevents cleavage in favor of transcriptional repression. The REC3 clamp is conserved across type II-B CRISPR–Cas9 systems, pointing to a potential path for engineering precise genome editors or developing novel antibacterial strategies. These findings reveal the molecular basis of heightened specificity and virulence enabled by FnCas9, with broad implications for biotechnology and therapeutic development.

## Graphical Abstract

**Graphical Abstract:**
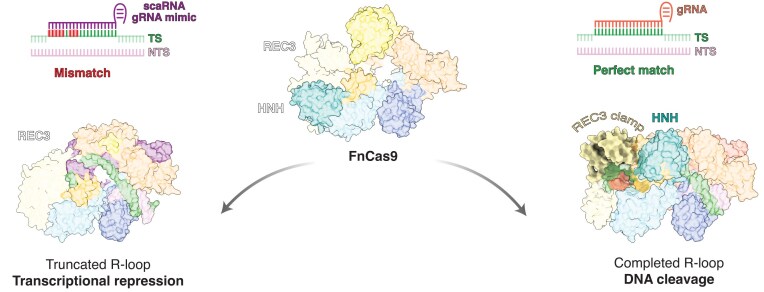

## Introduction

Bacterial and archaeal species have evolved CRISPR–Cas systems as an adaptive immune defense against foreign genetic elements, including bacteriophages and plasmids [1]. Among these systems, the Cas9 endonuclease quickly gained attention for its widespread applications in genome engineering [2–4]. *Streptococcus pyogenes* Cas9 (SpCas9) is the most used Cas9 ortholog, but suffers from off-target effects, which pose the risk of introducing unintended genomic modifications [5, 6]. Multiple groups have attempted to improve SpCas9 fidelity through directed evolution and rational engineering, often trading off cleavage efficiency for reduced off-target activity [7–11].

SpCas9 has been repurposed for widespread applications beyond genome editing, including targeted transcriptional regulation [12–14]. To accomplish transcriptional repression, the catalytic residues of SpCas9 are mutated to produce a catalytically “dead” Cas9 (dCas9) that binds DNA without the possibility of cleavage. By fusing dCas9 to transcriptional effectors, one can direct the complex to promoter or enhancer regions for gene silencing or activation, respectively [15, 16]. This approach ensures that the cleavage activity is eliminated by preventing any unintentional genomic damage.

In the search for naturally occurring high-fidelity alternatives, *Francisella novicida* Cas9 (FnCas9) emerged as an enzyme with intrinsic specificity [17]. Unlike SpCas9, which tolerates mismatches at the distal end of the RNA–DNA heteroduplex [18, 19], FnCas9 shows dramatically reduced cleavage in the presence of these same mismatches. Although early studies demonstrated this enhanced specificity, the molecular basis remains poorly understood.

Beyond its role in genome editing, *F. novicida* uniquely encodes a small CRISPR-associated RNA (scaRNA) that directs FnCas9 to repress, rather than cleave, an endogenous bacterial lipoprotein gene [20, 21]. This transcriptional silencing mechanism aids *F. novicida* in evading host immune detection. Crucially, FnCas9 achieves transcriptional repression while retaining cleavage activity. FnCas9 can have dual function as both a DNA endonuclease and a transcriptional repressor in its native, catalytically active form. This is in stark contrast to SpCas9, which requires catalytic inactivation to safely mediate transcriptional control without inadvertently cleaving target DNA.

The dual functionality of FnCas9 hints at a unique mechanistic checkpoint enabling the enzyme to discriminate between targets destined for cleavage and those targeted for transcriptional repression. Here, we combine kinetic measurements of mismatch discrimination with cryo-electron microscopy (cryo-EM) structural analysis to capture distinct FnCas9 conformational states. From these data, we identify a previously unrecognized structural feature that serves as a checkpoint in FnCas9 activation that we call the REC3 clamp. We demonstrate that nearly full R-loop complementarity is required for the REC3 clamp to dock and trigger DNA cleavage. In contrast, incomplete complementarity prevents REC3 clamp docking and thereby represses transcription as visualized by cryo-EM structures of FnCas9 with scaRNA-bound targets. Thus, our findings reveal how FnCas9 accomplishes high-fidelity DNA cleavage and selective transcriptional repression.

Finally, we establish that the REC3 clamp is highly conserved across type II-B CRISPR–Cas9 enzymes, suggesting that this dual function may be a hallmark of related Cas9 orthologs. By detailing the structural and mechanistic underpinnings of FnCas9 specificity and dual functionality, our work provides a blueprint for developing high-fidelity genome editors and leveraging the scaRNA-based repression mechanism for antibacterial strategies.

## Materials and methods

### Protein expression and purification

FnCas9 was expressed from pET-His6-FnCas9GFP purchased from Addgene (Addgene plasmid #130966; http://n2t.net/addgene:130966; RRID:Addgene_130966) [17]. FnCas9 was then expressed and purified as previously described [17], except NiCo21(DE3) (New England Biolabs) was used instead of Rosetta2 (DE3). Additionally, after affinity purification with Ni-NTA beads, the lysate was exposed to TEV protease to liberate GFP. The remainder of the purification was executed as previously described.

FnCas9 Y794A mutant was cloned using Q5 Hot Start Hi-Fidelity polymerase and KLD kit (New England Biolabs). The mutated sequences were verified by Plasmidsaurus and purified in the same manner as wild-type FnCas9.

### Nucleic acid preparation

DNA duplexes 55 nt long were prepared from polyacrylamide gel electrophoresis-purified oligonucleotides synthesized by Integrated DNA Technologies, as previously described [22]. The sequences of the synthesized oligonucleotides, including the positions of mismatches, are listed in Supplementary Table S2.

### Buffer composition and kinetic reactions

Cleavage reactions were performed in 1× cleavage buffer (20  mM Tris–Cl, pH 7.5, 100  mM KCl, 10 mM MgCl_2_, 5% glycerol, 1 mM dithiothreitol (DTT)) at 37°C.

### DNA cleavage kinetics

The reaction of FnCas9 with on- and off-target DNA was performed by preincubating FnCas9–gRNA [500 nM active-site concentration of FnCas9 and 750 nM guide RNA (gRNA)] in 5× cleavage buffer supplemented with 0.2 mg/ml molecular biology grade bovine serum albumin for 10 min at room temperature. The reaction was initiated by the addition of 5′-FAM-labeled DNA duplex at the final concentrations of 10 nM DNA and 100 nM preincubated enzyme.

The reactions were carried out at 37°C and stopped at various times by mixing with 0.5 M ethylenediaminetetraacetic acid. Reaction products were resolved and quantified using an Applied Biosystems DNA sequencer (ABI 3130xl) equipped with a 36-cm capillary array and nanoPOP7 polymer (MCLab) [22]. Data fits to equations were fit using either a single- or double-exponential equation shown below.

Single-exponential equation:


\begin{eqnarray*}
Y = {A_1}{{\rm e}^{ - {\lambda _1}t}} + C,
\end{eqnarray*}

where *Y* represents the concentration of cleavage product, *A*_1_ represents the amplitude, λ_1_ represents the observed decay rate (eigenvalue), and *C* is the endpoint. The half-life was calculated as *t*_1/2_ = ln(2)/λ_1_.

Double-exponential equation:


\begin{eqnarray*}
Y = {A_1}{{\rm e}^{ - {\lambda _1}t}}{A_2}{{\rm e}^{ - {\lambda _2}t}} + C,
\end{eqnarray*}

where *Y* represents the concentration of cleavage product, *A*_1_ represents the amplitude, and *λ*_1_ represents the observed rate for the first phase. *A*_2_ represents the amplitude, *λ*_2_ represents the observed rate for the second phase, and *C* is the endpoint.

### Cryo-EM sample preparation, data collection, and processing

FnCas9 bound to different DNA substrates was assembled by mixing FnCas9 with gRNA in a 1:1.5 molar ratio and incubated at room temperature for 10 min in reaction buffer (20  mM Tris–Cl, pH 7.5, 100  mM KCl, 10  mM MgCl_2_, 5% glycerol, and 5  mM DTT). Each DNA substrate was then added in a 1:1 molar ratio with either 8  μM FnCas9 gRNP for the on-target DNA substrate or 10 μM FnCas9 gRNP for the off-target DNA substrate and scaRNA 1101 DNA substrate. The on-target complex was incubated for 1 h at room temperature, and the off-target complex was incubated for 2 h at room temperature. The reactions were quenched by vitrification. 2.5  μl of sample was applied to glow-discharged holey carbon grids (Quantifoil 1.2/1.3), blotted for 6 s with a blot force of 0, and rapidly plunged into liquid nitrogen-cooled ethane using an FEI Vitrobot Mark IV.

FnCas9 perfect match and mismatch datasets were collected on an FEI Titan Krios cryo-electron microscope equipped with a K3 Summit direct electron detector (Gatan, Pleasanton, CA). Images were recorded with SerialEM v4.1 [23] with a pixel size of 0.83 Å. Movies were recorded at 13.3 electrons/pixel/s for 6 s (80 frames) to give a total dose of 80 electrons/pixel. FnCas9 scaRNA 1101 DNA dataset was collected on an FEI Glacios cryo-TEM equipped with a Falcon 4 detector with a pixel size of 0.933 Å, and a total exposure time of 15 s resulting in a total accumulated dose of 49 e/Å^2^. All datasets were collected with a defocus range of −1.5 to −2.5  μm. Motion correction, CTF estimation, and particle picking were performed on the fly using cryoSPARC Live v4.0.0-privatebeta.2 [24]. Further data processing was performed with cryoSPARC v.3.2. A total of 5378 movies were collected for the on-target dataset, 7158 movies were collected for the off-target dataset, and 2583 movies were collected for the scaRNA dataset.

Data processing mimicked workflows previously established for heterogeneous cryo-EM datasets [25]. The initial stages of processing were similar for all datasets, where blob picker was used with a minimum particle diameter of 120 Å and a maximum particle diameter of 220 Å. The off-target dataset particles were then subjected to a single round of 2D classification. The particles selected from 2D classification were processed via *ab initio* reconstruction, followed by heterogeneous refinement. The on-target and scaRNA dataset particles were not subjected to 2D classification, but rather went straight into *ab initio* reconstruction, followed by heterogeneous refinement. The best class from heterogeneous refinement was then fed to nonuniform refinement. The mask from the nonuniform refinement was used for 3D variability, which was displayed using the cluster output mode, with 10 clusters, and a 5 Å filter resolution. The clusters were grouped by the presence of the HNH, RuvC, and REC3 domains for the on-target dataset. The particles corresponding to a single state were extracted with a 400-pixel box size for the on-target particles, 420-pixel box size for the scaRNA particles, and 384-pixel box size for the off-target particles. The extracted particles were then globally and locally CTF refined, and fed to nonuniform refinement, which yielded the final reconstructions.

### Structural model building and refinement

Inactive FnCas9 [Protein Data Bank (PDB) code: 5B2O] was used as a starting model for the on-target FnCas9 structure with RuvC and HNH present. The HNH domain from the starting model was removed and replaced through rigid body fitting with a model of the HNH domain folded by AlphaFold2 [26]. HNH L1 and L2 were built, and nucleic acid alterations were made in Coot v1.1.07 [27]. Further modeling was performed using Isolde v1.6 [28]. The model was ultimately subjected to real-space refinement implemented in Phenix v1.21 [29]. The remaining on-target structures used the FnCas9 structure with RuvC and HNH present as a starting model. The productive off-target structure also used the FnCas9 structure with RuvC and HNH present as a starting model, but the nonproductive off-target structure used the nonproductive structure of FnCas9 with RuvC present as its starting model. The Cas9 sequence from *F. novicida U112* was supplied to the AlphaFold3 web server [30], along with a gRNA mimicking scaRNA and a 72-bp DNA comprising the 5′UTR of *FTN_1101*. This predicted model was used as a starting model for the scaRNA gRNA 1101 DNA structure. These structures were then subjected to a similar refinement workflow, where nucleic acid alterations were made in Coot v1.1.07 [27], modeling was done in Isolde v1.6 [28], and ultimately subjected to real-space refinement implemented in Phenix v1.21 [29]. All structural figures were generated using ChimeraX v1.2 [31].

### Conservation analysis

The on-target FnCas9 structure with RuvC and HNH present was given to ConSurf [32] for conservation analysis. Homologues were collected from the UniProt [33] database via an HMMR search [34]. The *E*-value cutoff was set to 0.001 with three iterations. The CD-HIT cutoff was set to 99%, maximum number of homologues to 150, and minimum sequence identity to 10%. The minimum query sequence coverage was set to 60%. A multiple sequence alignment was then built using MAFFT, and conservation scores were calculated by the Bayesian method [35]. The conservation scores were then mapped onto the on-target FnCas9 structure with RuvC and HNH present. The bar graph depicting amino acid position versus conservation score was made using Jalview [36].

## Results

### FnCas9 undergoes distinct structural changes during nuclease activation

**Figure 1.**
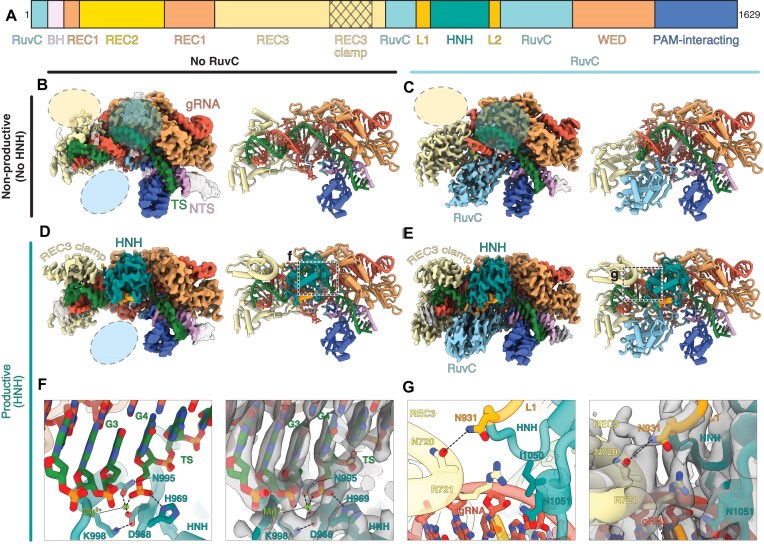
Visualization of distinct stages of FnCas9 nuclease activation. (**A**) FnCas9 domain organization with the REC3 clamp crosshatched. (**B**) 2.9 Å cryo-EM reconstruction of FnCas9 in a nonproductive state with no RuvC density resolved. Ovals indicate missing cryo-EM density. The corresponding models are depicted to the right of the cryo-EM reconstructions. (**C**) 2.9 Å cryo-EM reconstruction of FnCas9 in a nonproductive state with RuvC density. (**D**) 3.0 Å cryo-EM reconstruction of FnCas9 in a productive state with HNH in the active conformation and no RuvC density. (**E**) 2.6 Å cryo-EM reconstruction of FnCas9 in a productive state with RuvC density. (**F**) Detailed view of the HNH active site with the cryo-EM map overlaid. (**G**) Detailed view of the REC3 clamp and HNH L1 interactions with the cryo-EM map overlaid.

Previous structural studies of FnCas9 were performed using inactive enzyme, where the catalytic residue N995 in the HNH domain was mutated to alanine [37]. Inactivating HNH may hinder structural rearrangements required for enzyme activation [38, 39]. To elucidate the conformational changes associated with FnCas9 activation, we prepared cryo-EM grids of fully active FnCas9 in complex with gRNA and 55-bp target DNA after a 1-h incubation. The target DNA substrate contained the preferred PAM for FnCas9, NGG [37]. This single cryo-EM dataset yielded four unique, high-resolution reconstructions of FnCas9 during the process of enzyme activation (Fig. 1, Supplementary Figs S1 and S2, and Supplementary Table S1). The reconstructions were distinguished by the presence or absence of the two catalytic domains, HNH and RuvC.

All four reconstructions demonstrate that FnCas9 recognizes the NGG PAM primarily through hydrogen bonding between G2 and R1585, and G3 and R1556 (Supplementary Fig. S3), consistent with previous reports [37]. The REC2 domain is disordered in all the reconstructions from this dataset due to flexibility in the active enzyme. The two nonproductive structures of FnCas9 do not contain resolvable HNH density, indicating that the HNH domain is flexibly tethered and inactive in these states. We also noticed that a large portion of REC3 is missing from the nonproductive reconstructions. In the nonproductive reconstruction where RuvC is observed, only 13 bp of the R-loop are resolved, in comparison to the 21 bp resolved in our productive conformations (Fig. 1C). At lower thresholds, the PAM-distal R-loop can be seen sticking out away from the enzyme, hence why it was averaged out in the high-resolution reconstruction (Supplementary Fig. S4). In the other nonproductive structure without RuvC density, we observe the R-loop in its entirety, including contacts formed between REC3 and the PAM-distal R-loop (Fig. 1B and Supplementary Fig. S5). REC3 residue Q717 is inserted into the minor groove of the heteroduplex between positions 15 and 16. Q801 contacts the gRNA phosphate backbone at position 15 of the R-loop. N812 similarly contacts the target strand (TS) phosphate backbone, but between R-loop positions 17 and 18. These contacts anchor REC3 to the R-loop, stabilizing its PAM-distal end and facilitating the conformational shift of HNH into a cleavage-competent state. Together, the nonproductive structures of FnCas9 point to a checkpoint during enzyme activation where REC3 residues 710–801 are disordered prior to docking onto the PAM-distal heteroduplex. We call this portion of REC3 the REC3 clamp.

The productive FnCas9 reconstructions from this dataset contain well-resolved HNH domains in the active conformation (Fig. 1D and E). Furthermore, the phosphodiester backbone of the TS is cleaved at the anticipated HNH endonuclease site three nucleotides upstream of the PAM (Fig. 1F). The REC3 clamp is also well resolved and forms stabilizing contacts with the completed R-loop and linker 1 of the HNH domain (L1) (Fig. 1G). Specifically, N720 in the REC3 clamp and N931 in L1 form a hydrogen bond, relaying REC3 engagement toward catalytic activation. In the productive structure lacking RuvC density, we observe flexibility in the portion of REC3 outside of the clamp that typically interfaces with RuvC, although the REC3 clamp remains in contact with the PAM-distal heteroduplex. These findings suggest a stepwise mechanism in which the REC3 clamp must dock onto the fully formed R-loop prior to HNH rearrangement and subsequent DNA cleavage.

### The REC3 clamp confers FnCas9 specificity

**Figure 2:**
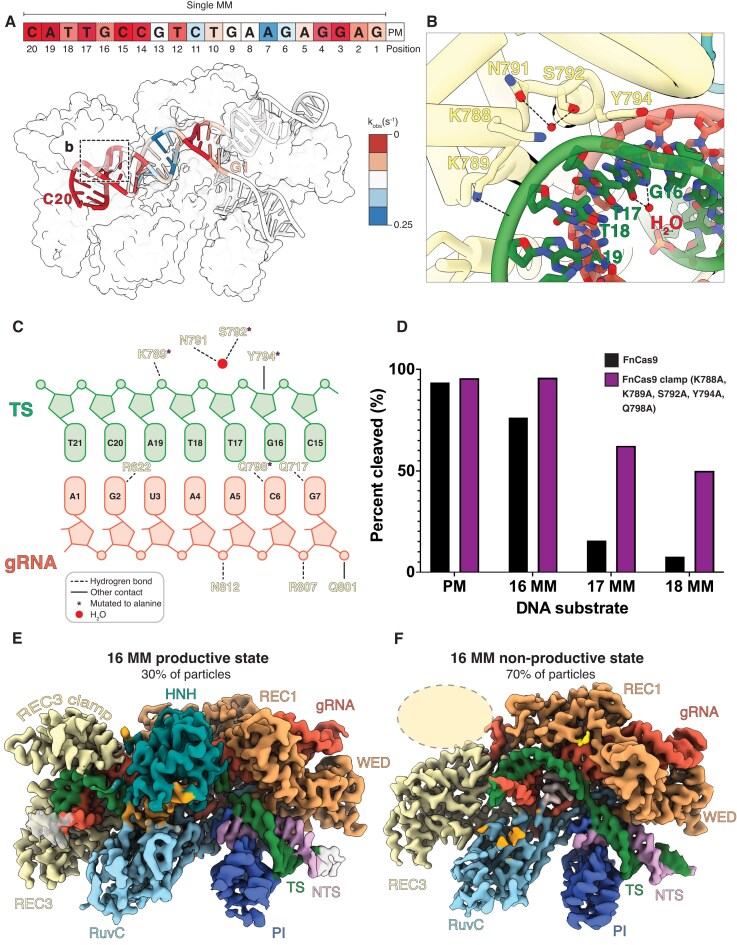
The REC3 clamp enhances FnCas9 specificity. (**A**) Surface representation of FnCas9 in the product state with the nucleotides colored by observed cleavage rate for a single mismatch at that TS position. PM represents a perfect match DNA substrate. Observed cleavage rates were measured for each mismatch via capillary electrophoresis. Red indicates a slower observed cleavage rate, and blue indicates a faster observed cleavage rate. (**B**) Detailed view of REC3 clamp residues contacting the PAM-distal heteroduplex in the product state conformation. (**C**) Schematic representation of REC3 contacts with the PAM-distal heteroduplex. Dashed lines represent hydrogen bonds; solid lines represent other contacts; asterisks represent the residues mutated to alanine; red circles represent water molecules. (**D**) 100 nM of each FnCas9 variant was mixed with 25 nM of each substrate, and the cleaved DNA product was monitored via capillary electrophoresis. Percent cleaved reported after 1 h incubation. Black denotes wild-type FnCas9; purple denotes the FnCas9 clamp mutant. (E, F) FnCas9 was incubated with DNA containing a mismatch at position 16 of the TS for 1 h. The reaction was quenched via vitrification and analyzed via cryo-EM. (**E**) Thirty percent of the particles (79,858 particles) from this dataset yielded a 2.9 Å cryo-EM reconstruction of FnCas9 in a productive state bound to DNA with a mismatch at position 16. (**F**) Seventy percent of the particles (128,469 particles) from this dataset comprised a 3.0 Å cryo-EM reconstruction of FnCas9 in a nonproductive state bound to DNA with a mismatch at position 16.

SpCas9 is well documented to tolerate mismatches in the PAM-distal region, leading to cleavage of off-target sequences, which pose a significant challenge in genome editing applications [5, 10, 40, 41]. In contrast, FnCas9 exhibits a markedly higher sensitivity to mismatches in this region, where a single mismatch can abolish cleavage activity [17]. While this intrinsic specificity has been observed, the underlying molecular mechanism has remained elusive. To investigate this, we analyzed the cleavage kinetics of FnCas9 using gRNA and DNA substrates containing a single mismatch at each position along the protospacer (Fig. 2A and Supplementary Table S2).

Our results revealed that mismatches in the PAM-distal region slowed cleavage rates by up to 155-fold compared to perfectly matched substrates (Fig. 2A and Supplementary Table S3). Structural inspection of the productive state of FnCas9 uncovered extensive contacts between the REC3 clamp and the PAM-distal heteroduplex, particularly in regions where mismatches significantly impaired cleavage (Fig. 2B and C). These interactions include N791 and S792, which coordinate a water molecule with the TS phosphodiester backbone, and K789, which forms electrostatic interactions with the TS backbone at positions 18 and 19. Most notably, Y794 interacts with the ribose at position 16 of the TS, stabilizing the PAM-distal region (Fig. 2B and C, and Supplementary Fig. S5).

We next evaluated the functional importance of REC3 clamp residues K788, K789, S792, Y794, and Q798 by mutating each to alanine. These mutations disrupted critical contacts between the REC3 clamp and the PAM-distal heteroduplex, resulting in reduced FnCas9 specificity for mismatches in this region. Compared to wild-type FnCas9, the K788A, K789A, S792A, Y794A, and Q798A FnCas9 clamp mutant exhibited increased cleavage of PAM-distal mismatches after a 1 h incubation (Fig. 2D and Supplementary Fig. S6). These results confirm that K788, K789, S792, Y794, and Q798 are essential for stabilizing the interaction of the REC3 clamp with the PAM-distal duplex, thereby contributing to FnCas9 mismatch discrimination and cleavage efficiency.

To probe the functional relevance of Y794, we performed cryo-EM studies of FnCas9 with gRNA and a DNA substrate with a mismatch at position 16 of the protospacer. These structural studies revealed that only ∼30% of particles adopted a productive state, where the HNH domain had rearranged into the catalytically active conformation, while the remaining ∼70% of particles were trapped in a nonproductive state (Fig. 2E and F, and Supplementary Figs S7 and S8). This proportion of productive particles is far less than the ∼69% of productive particles with a perfectly matched substrate. Despite the mismatch, Y794 maintained its interaction with the TS in the productive particles, highlighting the importance of this residue in docking of the REC3 clamp.

Unlike SpCas9, which frequently tolerates mismatches in the PAM-distal region, FnCas9 relies on the REC3 clamp to dock onto the final base pairs of the R-loop. This unique mechanism underpins off-target stringency and provides a framework for engineering Cas9 variants with enhanced fidelity.

### Structural basis of heightened virulence in *F. novicida*

**Figure 3.**
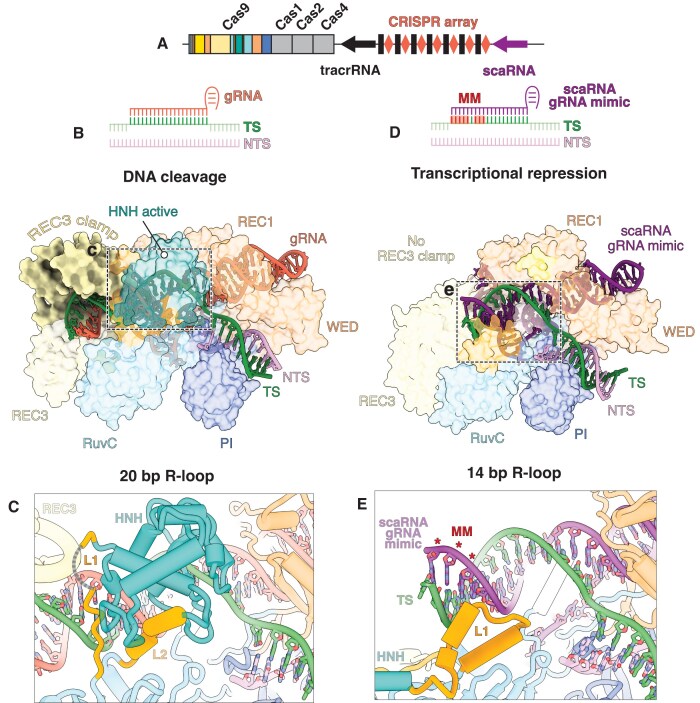
The REC3 clamp senses target complementarity distinguishing DNA cleavage from transcriptional repression. (**A**) Schematic of the *F. novicida* chromosomal locus consisting of Cas genes, tracrRNA, crRNA, and scaRNA. (**B**) Schematic of canonical FnCas9 targeting where 20 bp of R-loop form between the gRNA and TS. (**C**) Detailed view of L1 when the R-loop has propagated to completion and HNH is in the active conformation. (**D**) Schematic of scaRNA-mediated targeting where 11 bp of complementarity exists prior to encountering mismatches. 2.8 Å cryo-EM reconstruction of scaRNA-mediated targeting by FnCas9 with 14 bp of R-loop formed. (**E**) Detailed view of L1 in scaRNA-mediated transcriptional repression structure with mismatches shown in red.

Unlike canonical CRISPR–Cas9 systems that target foreign nucleic acid for cleavage, *F. novicida* encodes an additional scaRNA capable of repressing its own genes rather than destroying them [20, 21]. In *F. novicida*, scaRNA forms a complex with FnCas9 and tracrRNA to bind an endogenous bacterial lipoprotein (BLP) gene (Fig. 3A and Supplementary Fig. S9). Notably, this scaRNA complex does not cleave the BLP gene—an event that would damage its own genome—but instead represses its expression by targeting the 5′UTR. This repression evades host Toll-like receptor 2 detection, reducing immune activation.

To clarify how FnCas9 distinguishes cleavage versus repression of a given target, we solved a cryo-EM structure of FnCas9 bound to a single gRNA comprised of the canonical gRNA scaffold and the scaRNA spacer that targets the 5′UTR of *FTN_1101*—one of the genes encoding the BLP (Fig. 3D and Supplementary Figs S10 and S11). Our dataset revealed a dominant conformation in which the R-loop extends for 14 base pairs with positions 12–14 mismatched (Fig. 3E). This partial R-loop fails to recruit the REC3 clamp, which in turn prevents the HNH domain from adopting a cleavage-competent conformation. We observed the HNH linker 1 (L1) folded in an inactive conformation (Fig. 3E), consistent with an R-loop that is distorted and incomplete [10, 42].

In comparison, a fully matched target promotes R-loop completion, permits REC3 clamp docking, and enables HNH domain rearrangement for DNA cleavage (Fig. 3C). Thus, our findings reveal that the REC3 clamp imposes an additional activation step, allowing FnCas9 to toggle between cleavage and repression depending on R-loop length. This mechanism underscores how FnCas9 can be repurposed to silence endogenous genes and thereby enhance bacterial virulence without compromising bacterial genome integrity.

### The REC3 clamp is conserved across type II-B CRISPR systems

**Figure 4.**
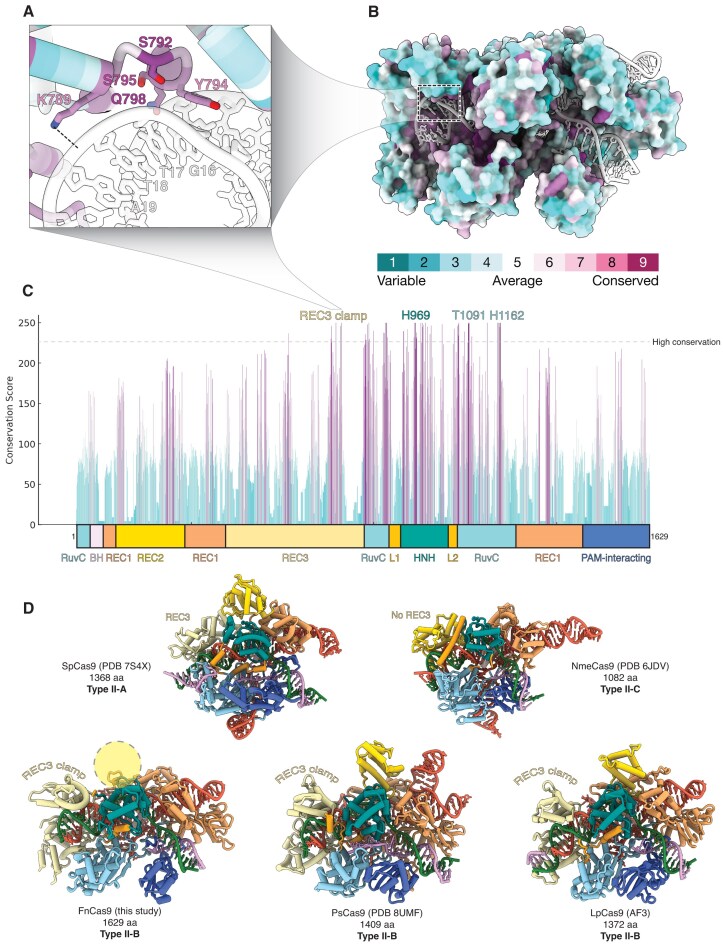
Type II-B CRISPR–Cas systems contain extended REC3 domains. (**A**) Detailed view of REC3 clamp residues contacting the PAM-distal heteroduplex colored by conservation score. The location of these residues is highlighted in panels (B) and (C). (**B**) Surface representation of FnCas9 in the product state where each residue is colored by conservation, where purple represents more conserved residues, and cyan denotes more variable residues. (**C**) Domain schematic of FnCas9 with the corresponding conservation score for each residue. Residues with a conservation score of 225 or higher are considered highly conserved. (**D**) Structural comparison of type II CRISPR–Cas9 enzymes, including type II-A SpCas9 (PDB 7S4X), type II-C NmeCas9 (PDB 6JDV), type II-B FnCas9 (this study), PsCas9 (PDB 8UMF), and LpCas9 (AF3). The REC3 domains are labeled for each structure. The REC2 domain is unresolved in the FnCas9 structure as indicated by the circle.

Type II-B CRISPR–Cas effectors are known to be larger than other type II enzymes [43], prompting us to question whether these systems share the extended REC3 domain observed in FnCas9, and possibly a similar mismatch-sensing mechanism. To address this question, we performed a homologue search across the UniProt [44] database using HMMER [34] and conservation analysis using ConSurf [32] of 51 type II-B Cas9 homologues (Supplementary Fig. S12) [44]. Our analysis revealed that the key REC3 clamp residues are as highly conserved as the catalytic residues in the HNH and RuvC domains (Fig. 4A–C). Notably, Q798, which intercalates between the gRNA and TS, S792, which coordinates a water molecule at the TS backbone, and Y794, which stacks on the target-strand ribose at position 16, all show strong sequence conservation.

In contrast, SpCas9 (a type II-A enzyme) contains a smaller REC3 domain (Fig. 4D and Supplementary Fig. S13), which lacks the extensive contacts seen in FnCas9 for sensing the final base pairs of the R-loop. This difference correlates with the established tolerance of SpCas9 for PAM-distal mismatches [5, 10, 40, 41]. Furthermore, type II-C enzymes such as Cas9 from *Neisseria meningitidis* (NmeCas9) contain no REC3 domain [38]. Instead, the REC2 domain of type II-C Cas9 adopts a similar conformation to the REC3 domain in other type II CRISPR–Cas systems, albeit with far less protein–nucleic acid interactions. When comparing type II CRISPR–Cas9 enzymes, the REC3 clamp emerges as a feature unique to type II-B. Other type II-B enzymes such as Cas9 from *Parasutterella secunda* (PsCas9) also contain extended REC3 domains with the clamp feature (Fig. 4D) [45].

One of the major drawbacks of genome editing with FnCas9 is its large effector, so we searched through FnCas9 homologues to identify a smaller type II-B Cas9 that may function with similarly high specificity. This search highlighted Cas9 from *Legionella pneumophila* (LpCas9), which is comprised of 1372 amino acids, effectively the same size as the widely used 1368 amino acid SpCas9 (Fig. 4D). We then predicted the structure of LpCas9 with the same HBB DNA target used for our FnCas9 studies and found that it appears to have a REC3 clamp with similar contacts formed with the PAM-distal heteroduplex as FnCas9. These findings indicate that LpCas9 strikes a balance between size and specificity.

**Figure 5.**
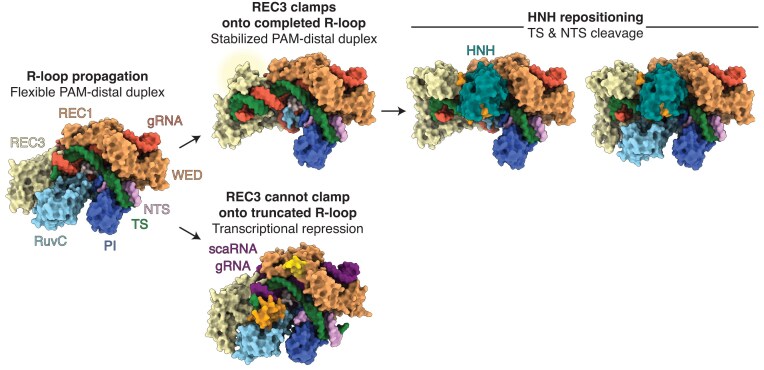
Model of type II-B CRISPR–Cas9 nuclease activation. If the R-loop propagates to completion, the REC3 clamp docks onto the PAM-distal heteroduplex prompting HNH repositioning and DNA cleavage. Alternatively, if REC3 cannot clamp due to incomplete R-loop formation, type II-B CRISPR–Cas9 repress transcription without cleavage.

Taken together, type II-B Cas9 enzymes contain extended REC3 domains capable of sensing PAM-distal mismatches, thereby imposing an additional activation step that minimizes off-target cleavage (Fig. 5). In *F. novicida*, the REC3 clamp also underlies the ability of the enzyme to repress rather than cleave specific endogenous genes, thus facilitating immune evasion through subverted host detection. Altogether, the REC3 clamp emerges as a hallmark of type II-B Cas9 systems, simultaneously driving heightened specificity and virulence. Its unique properties distinguish type II-B enzymes from other CRISPR–Cas systems and offer a promising framework for designing next-generation genome editors with improved fidelity and tunable outcomes.

## Discussion

Our work uncovers a novel specificity checkpoint in FnCas9, centered on the REC3 clamp, that transforms our understanding of how type II-B CRISPR–Cas9 orthologs achieve heightened fidelity. Distinct cryo-EM reconstructions reveal a stepwise activation process, where the R-loop must propagate to completion before the REC3 clamp docks, enabling HNH domain rearrangement and DNA cleavage. In contrast, SpCas9 is known to efficiently cleave DNA with mismatches, particularly in the PAM-distal region. This discrepancy arises because SpCas9 lacks a REC3 clamp, enabling L1 to dock onto the heteroduplex once the R-loop has propagated to ∼16 bp, thereby activating the HNH domain despite mismatches. This also underlies the rapid rate of SpCas9 DNA cleavage. However, in FnCas9, the REC3 clamp remains undocked if mismatches are present, preventing L1 from engaging the heteroduplex and halting HNH activation. This clamp mechanism explains why FnCas9 is so intolerant to PAM-distal mismatches—a common blind spot to SpCas9 (Supplementary Fig. S14) [10,46].

Notably, our identification of LpCas9, a compact type II-B Cas9 with a size comparable to SpCas9, demonstrates that the specificity-enhancing REC3 clamp can be retained in a smaller effector. Structural predictions of LpCas9 reveal a REC3 clamp with similar PAM-distal heteroduplex contacts as FnCas9, suggesting that LpCas9 offers a balance of high specificity and practical size for genome editing applications.

Beyond specificity, we also show how the REC3 clamp differentiates transcriptional repression from cleavage, evidenced by the scaRNA complex that targets the *F. novicida* chromosome. The REC3 clamp remains undocked when the R-loop is incomplete, leaving HNH in a nonproductive conformation. From a pathogenesis standpoint, this mechanism allows *F. novicida* to avoid host immune recognition by silencing critical BLP genes without risking self-inflicted genome damage.

The clamp and its high conservation among type II-B CRISPR–Cas9 enzymes have practical implications. First, the clamp domain could be engineered into other Cas9 variants or smaller Cas effectors to minimize off-target cleavage [47–50]. Second, the reliance of *F. novicida* and related pathogens on REC3 clamp-based repression suggests new antibacterial strategies, such as specifically targeting the clamp or scaRNA function to disrupt bacterial immune evasion and sensitize pathogens to immune clearance or antibiotics [20, 51, 52].

Overall, our findings provide a blueprint for advanced genome editing tools that harness the REC3 clamp for improved precision, with LpCas9 emerging as a promising platform for next-generation editors. They also highlight a previously underappreciated strategy in bacterial virulence that might be exploited to counter antibiotic resistance. As new CRISPR-based applications emerge, understanding the modular features that influence both cleavage and repression will be key to expanding our therapeutic and biotechnological toolkit.

## Supplementary Material

gkaf585_Supplemental_File

## Data Availability

The structures and associated atomic coordinates have been deposited into the Electron Microscopy Data Bank (EMDB) and Protein Data Bank (PDB) with accession codes FnCas9 productive (EMD-48052 and PDB 9EHF), FnCas9 productive no. RuvC (EMD-48053 and PDB 9EHG), FnCas9 nonproductive (EMD-48054 and PDB 9EHH), FnCas9 nonproductive no RuvC (EMD-48062 and PDB 9EHR), FnCas9 16 MM productive (EMD-48070 and PDB 9EHX), FnCas9 16 MM nonproductive (EMD-48069 and PDB 9EHW), and FnCas9 scaRNA gRNA 1101 DNA nonproductive (EMD-49074 and PDB 9N6T).
