# Supplementary material for: Structural basis of a dual-function type II-B CRISPR–Cas9
Source: Nucleic Acids Res. 2025 Jul 4;53(12):gkaf585. doi: 10.1093/nar/gkaf585 (PMC12231574; doi:10.1093/nar/gkaf585)
Supplement: gkaf585_Supplemental_File [file gkaf585_supplemental_file.pdf]

Supplementary Information

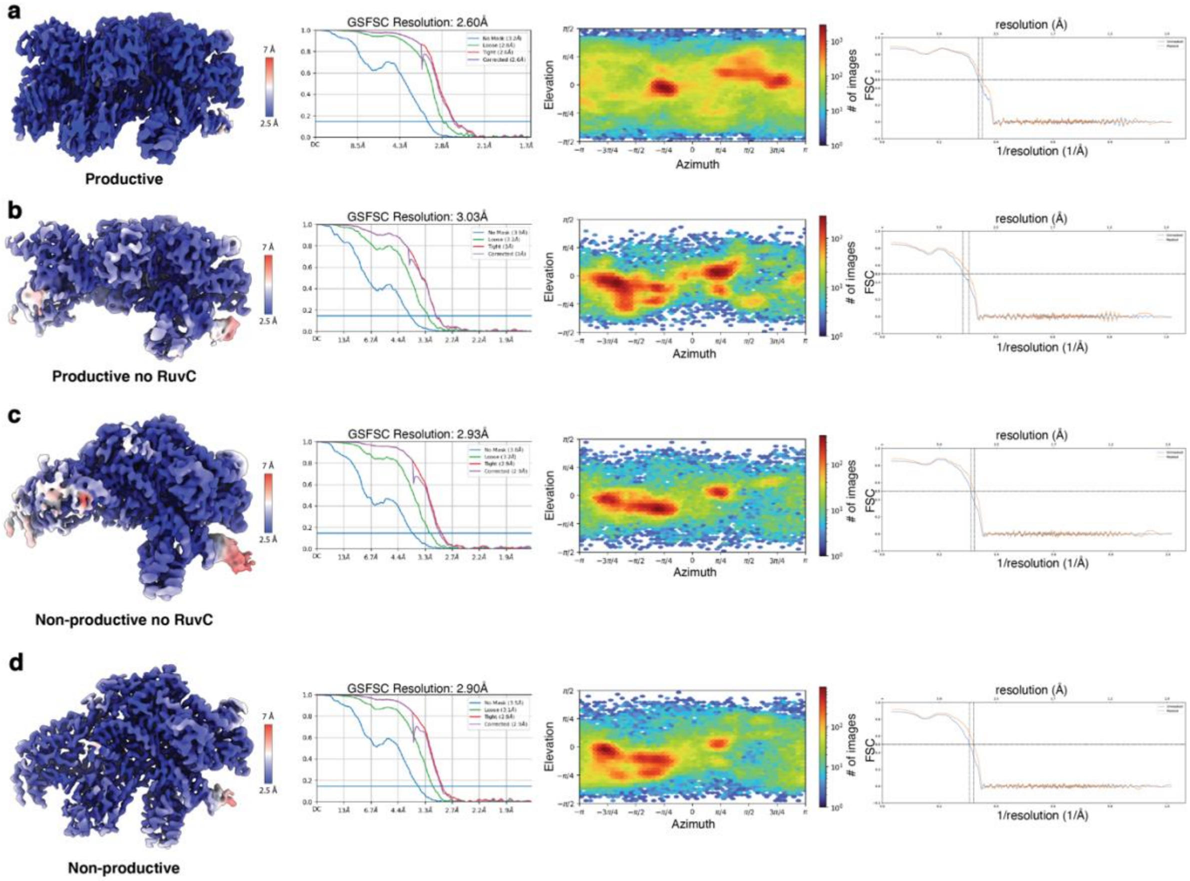

Supplementary Figure S1: Cryo-EM data analysis of FnCas9 bound to PM DNA in the a, productive b, productive no RuvC c, non-productive no RuvC and d, non-productive states.

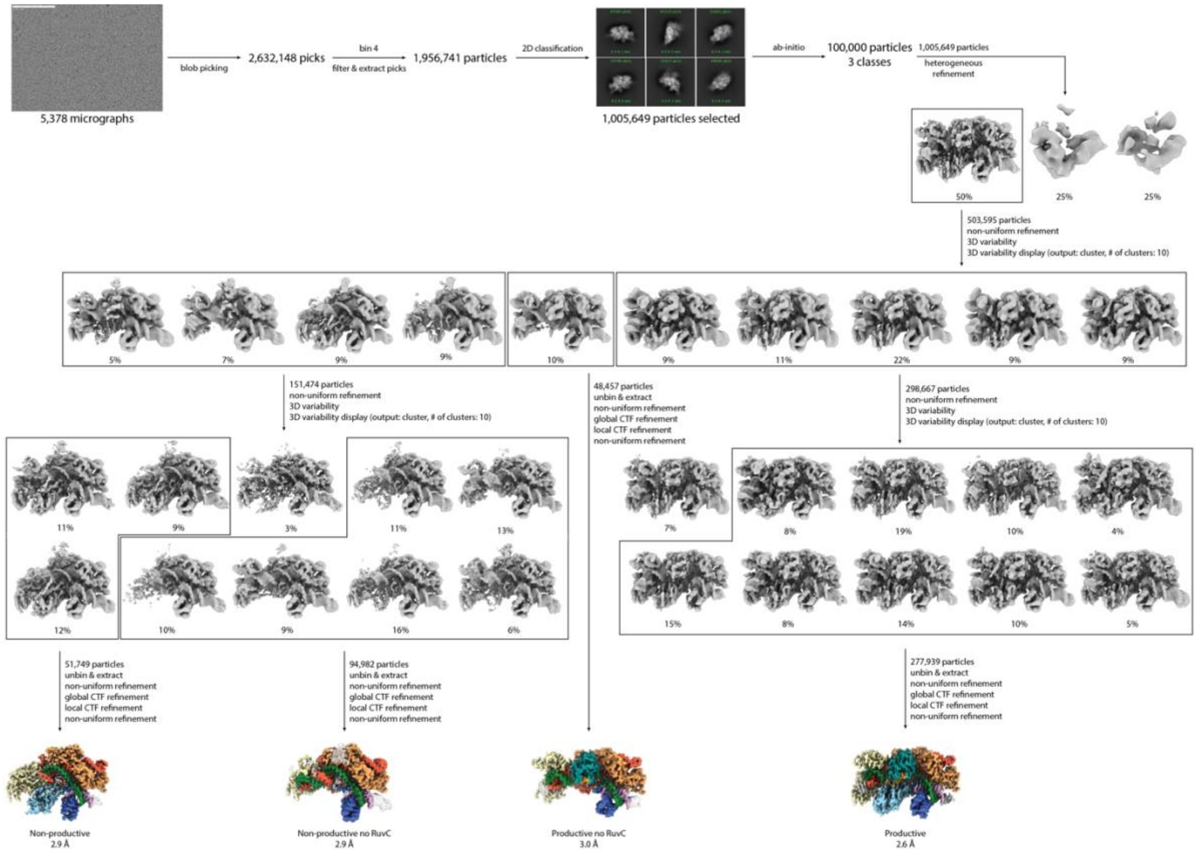

Supplementary Figure S2: Cryo-EM data processing workflow for FnCas9 bound to PM DNA after a 1-hour incubation at room temperature. Final reconstructions are colored by domain.

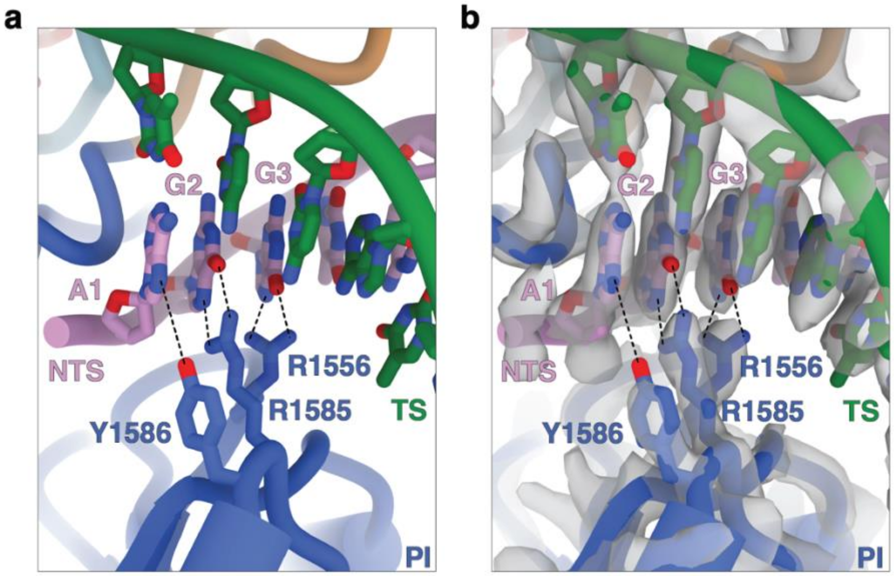

Supplementary Figure S3: FnCas9 residues R1556 and R1585 recognize the NGG PAM. a, Detailed view of the PAM site interaction when FnCas9 is in the product state. b, Cryo-EM density of the FnCas9 PAM site interaction.

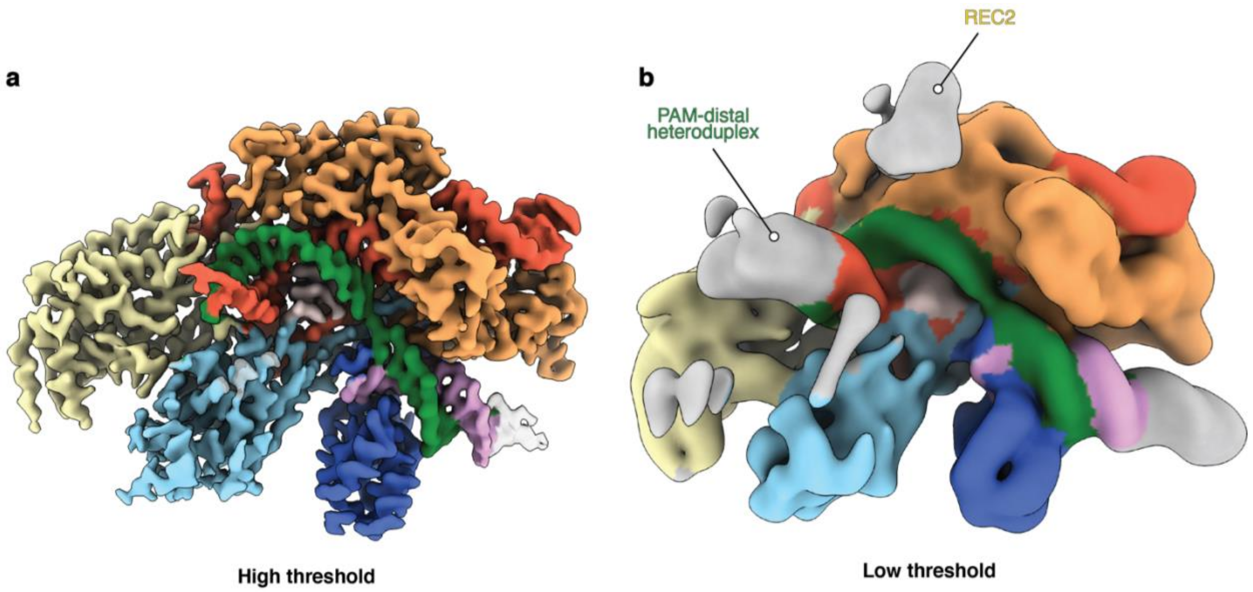

Supplementary Figure S4: The PAM-distal heteroduplex is flexible prior to REC3 docking. a, 2.9 Å cryo-EM reconstruction of FnCas9 in a non-productive conformation at a high threshold where the PAM-distal heteroduplex is averaged out, and b, at a low threshold where the PAM-distal heteroduplex is resolved.

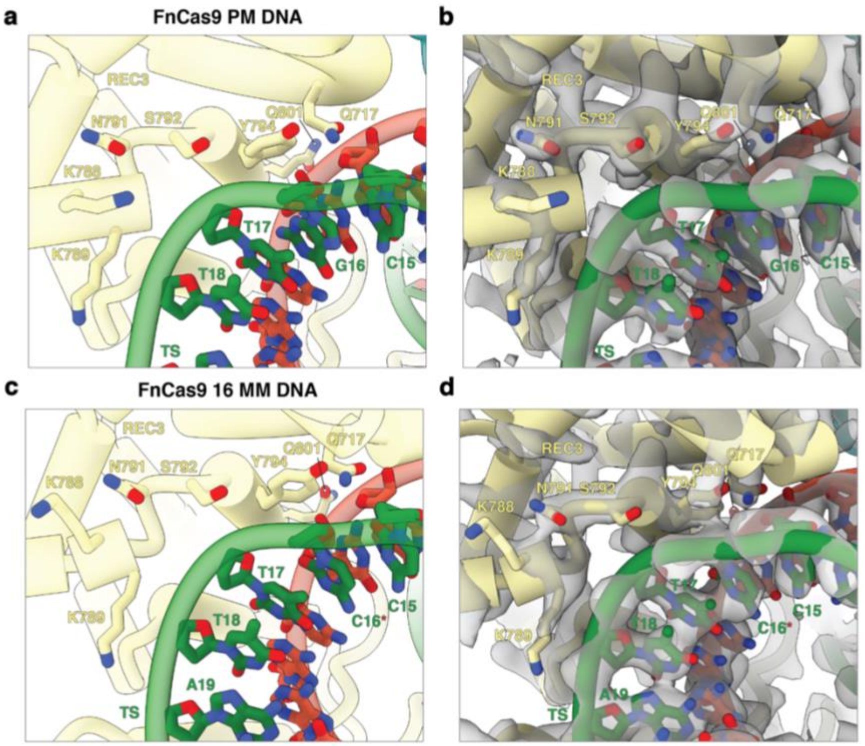

Supplementary Figure S5: REC3 clamp residues interact with the PAM-distal heteroduplex. a, Detailed view of the REC3 clamp contacts when FnCas9 is in complex with PM DNA in the product state. b, Cryo-EM density for the REC3 clamp contacts with PM DNA at 2.6 Å resolution. c, Detailed view of the REC3 clamp contacts when FnCas9 is in complex with 16 MM DNA in the product state. d, Cryo-EM density for the REC3 clamp contacts with 16 MM DNA at 2.9 Å resolution.

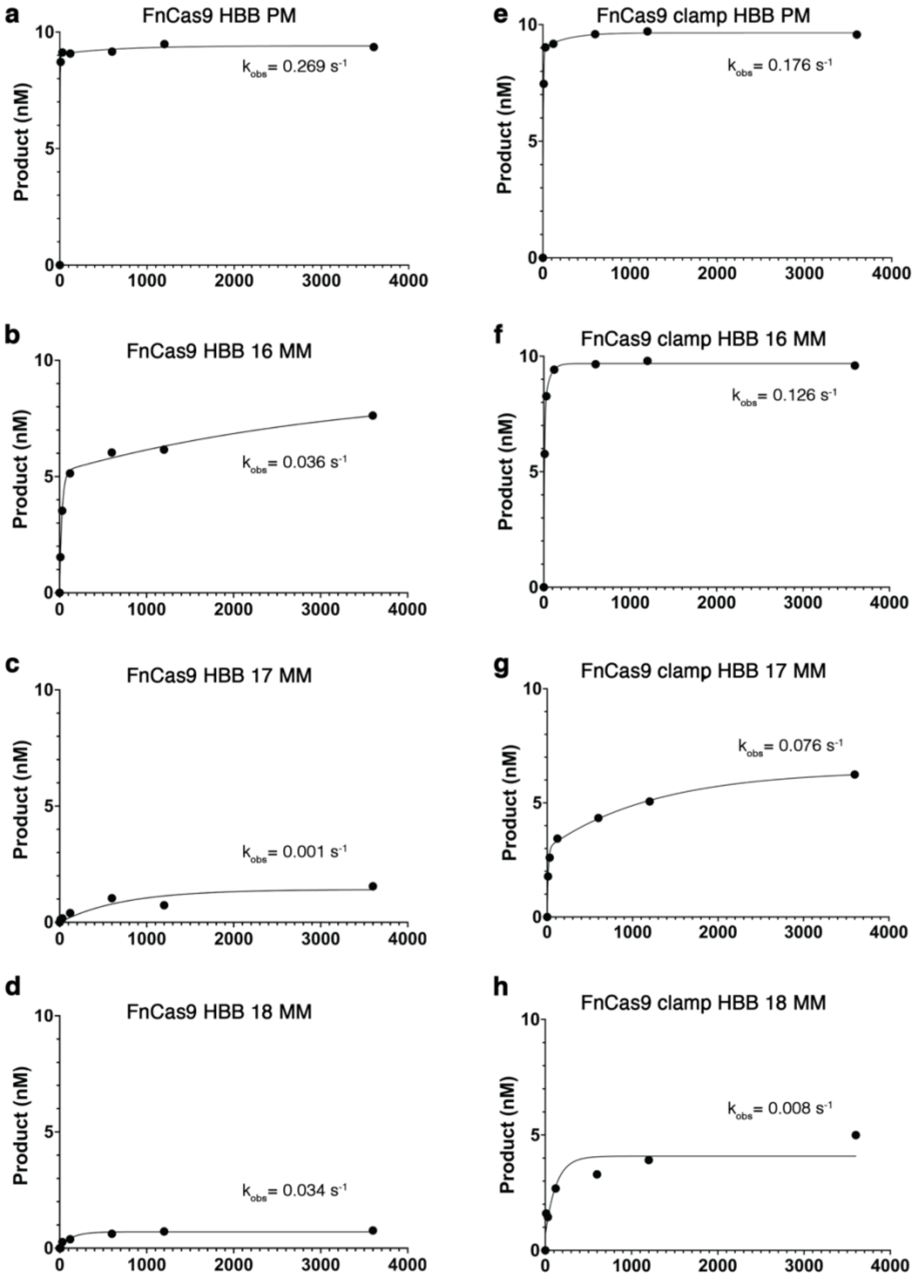

Supplementary Figure S6: Removing REC3 clamp contacts diminishes specificity. a-d, FnCas9 TS cleavage product formation over time for PM and PAM-distal MM DNA substrates. e-h, FnCas9 clamp mutant TS cleavage product formation over time for PM and PAM-distal MM DNA substrates. Observed cleavage rates are inlayed.

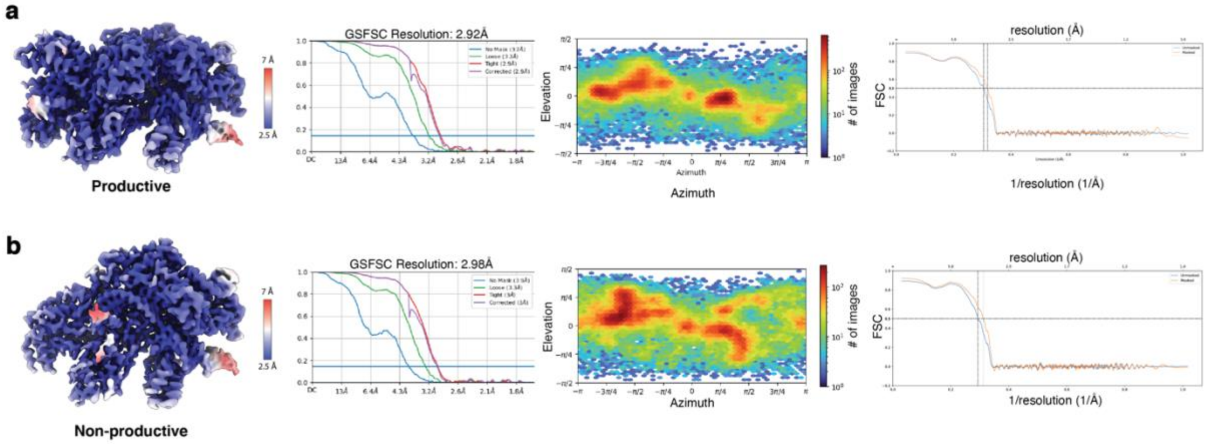

Supplementary Figure S7: Cryo-EM data analysis of FnCas9 bound to 16 MM DNA in the a, productive and b, non-productive states.

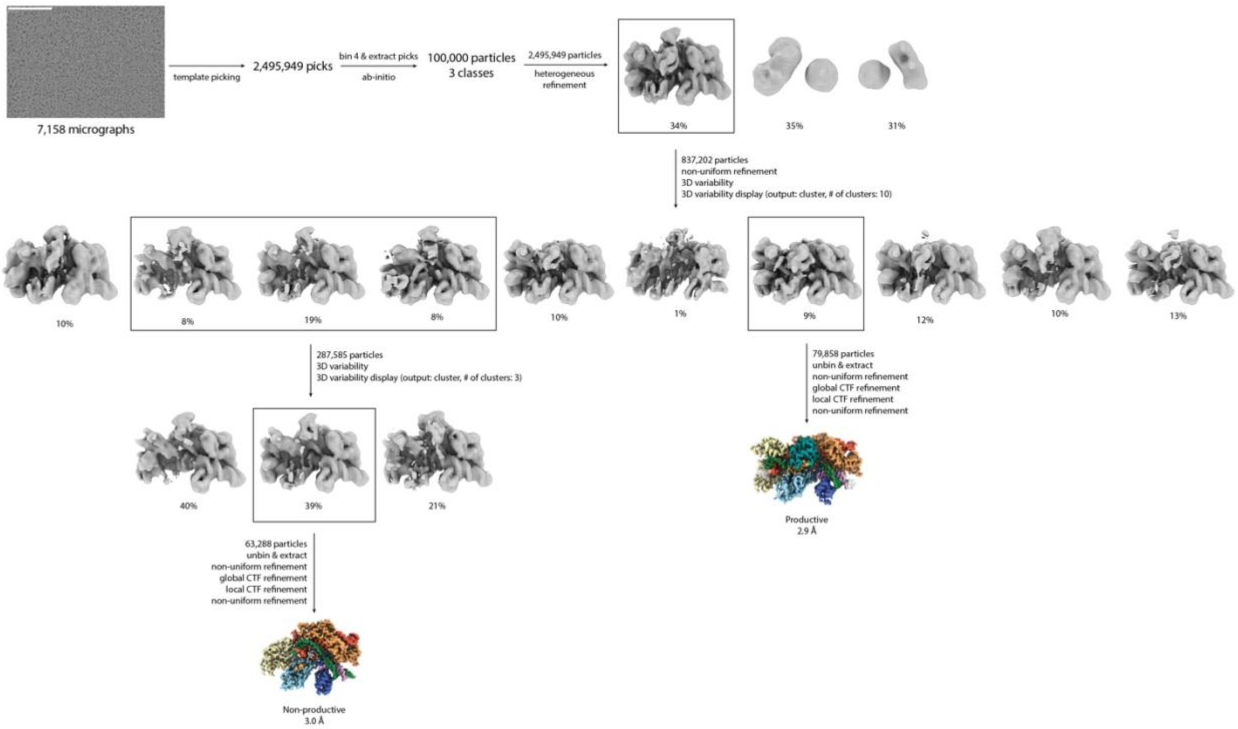

Supplementary Figure S8: Cryo-EM data processing workflow for FnCas9 bound to 16 MM DNA after a 2-hour incubation at room temperature. Final reconstructions are colored by domain.

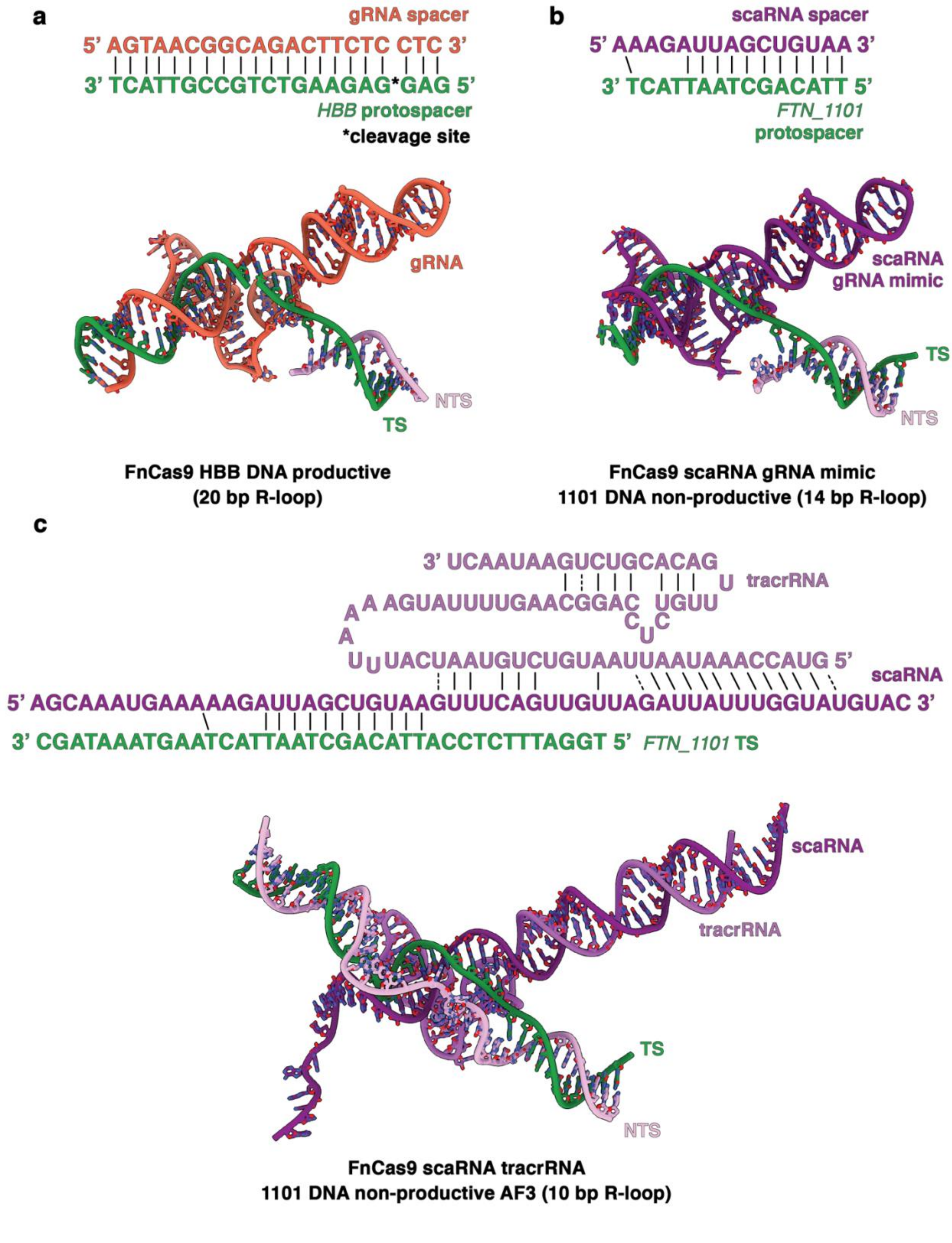

Supplementary Figure S9: Comparison between FnCas9 a, gRNA-, b, scaRNA gRNA mimic-mediated structures derived from cryo-EM, and c, scaRNA:tracrRNA-mediated

structure predicted by AF3. The sequences for each nucleic acid conformation are also provided.

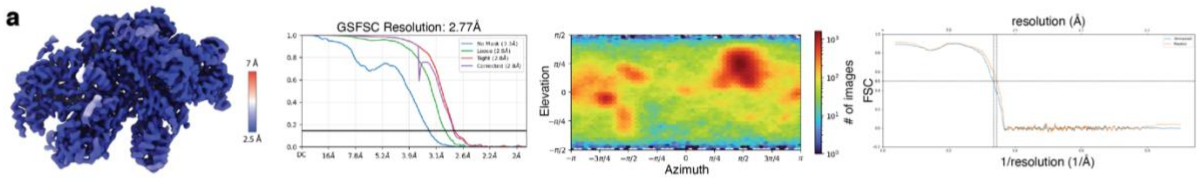

Supplementary Figure S10: Cryo-EM data analysis of FnCas9 bound to scaRNA gRNA targeting 1101 DNA in the a, non-productive state.

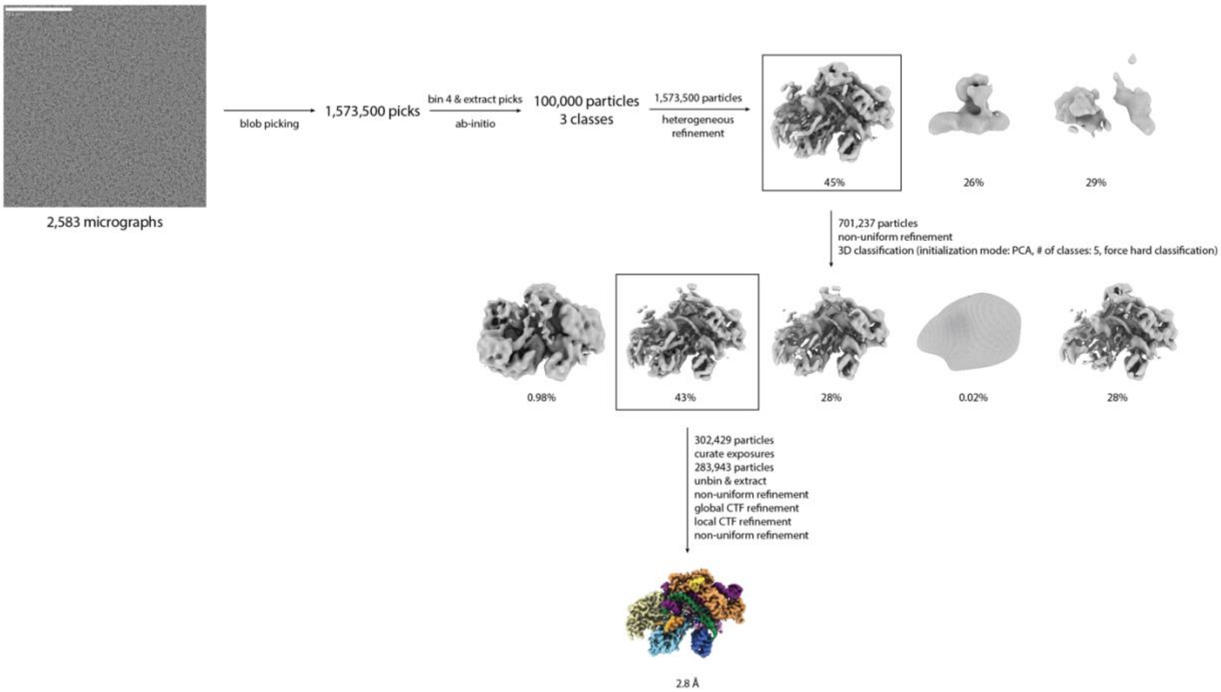

Supplementary Figure S11: Cryo-EM data processing workflow for FnCas9 bound to scaRNA-gRNA mimic and *FTN_1101* DNA after a 1-hour incubation at room temperature. Final reconstruction is colored by domain.

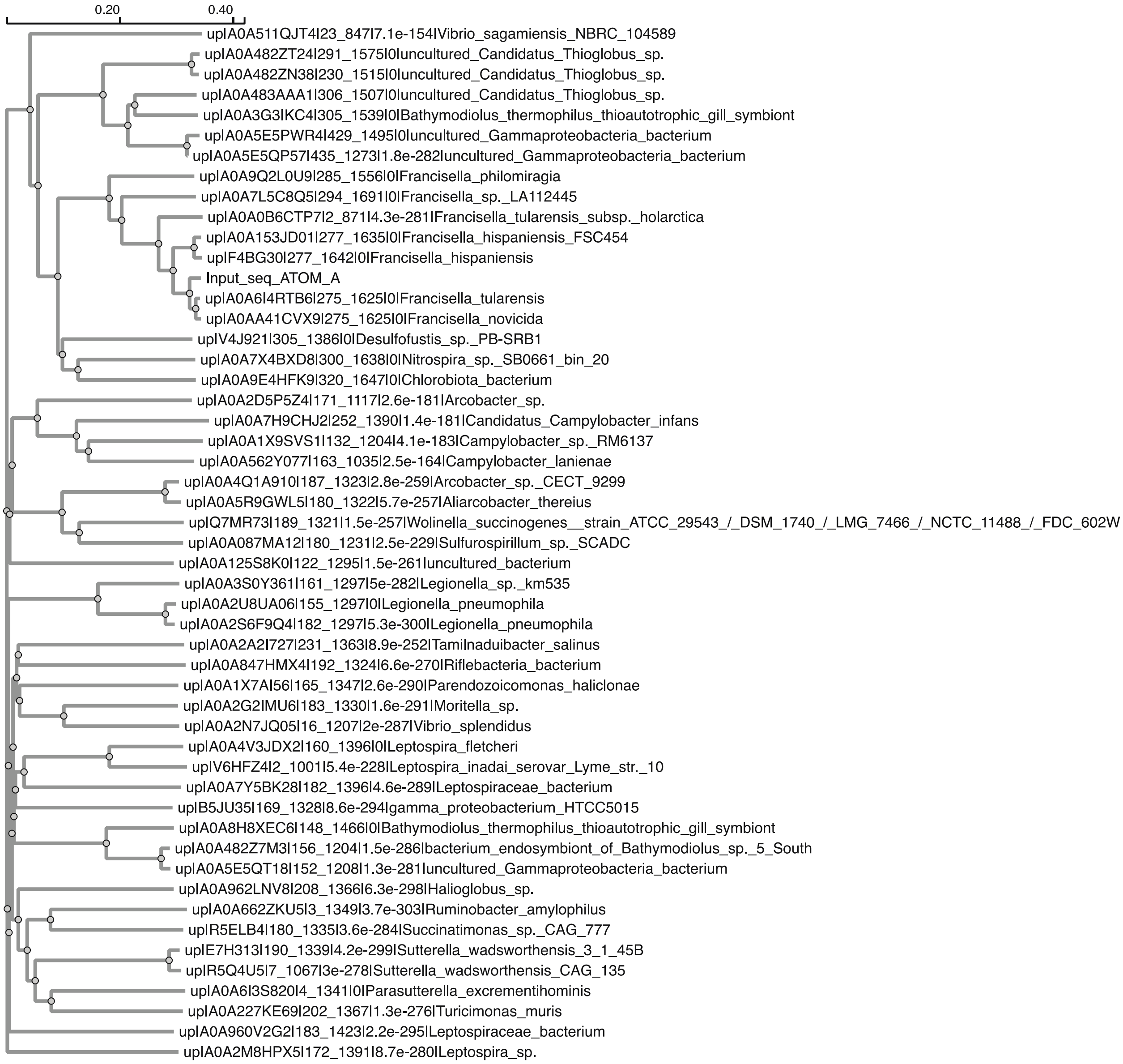

Supplementary Figure S12: Phylogenetic tree of type II-B Cas9 homologues used in conservation analysis with accession numbers [33].

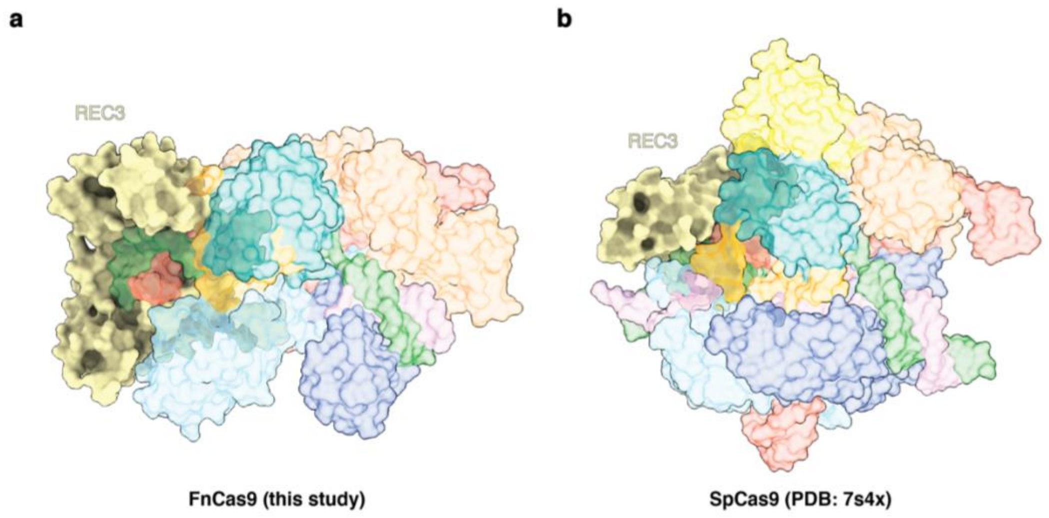

Supplementary Figure S13: FnCas9 and SpCas9 REC3 domain comparison. a, FnCas9 residues 450-858 comprise the REC3 domain. b, SpCas9 residues 496-718 comprise the REC3 domain. REC3 domains are opaque.

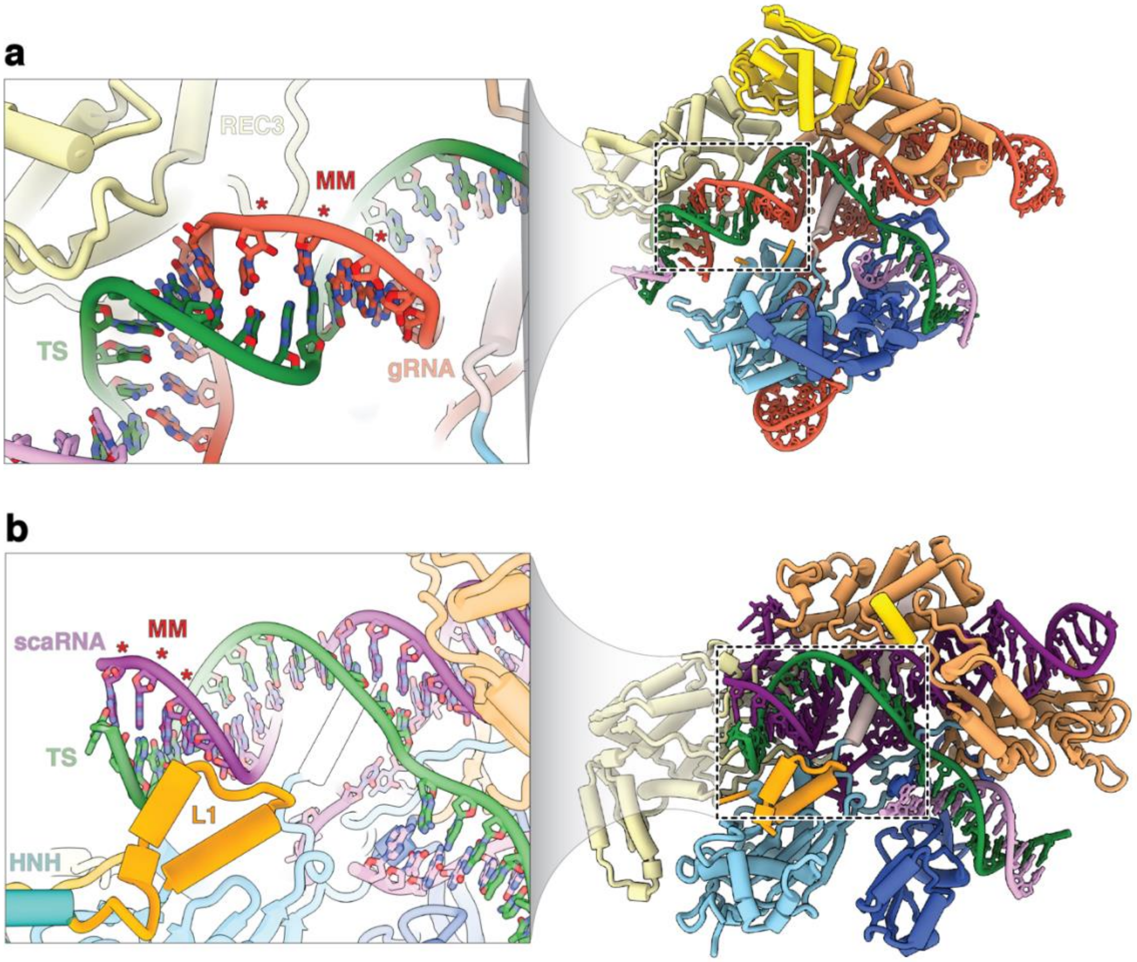

Supplementary Figure S14: Comparison of SpCas9 and FnCas9 12-14 MM surveillance structures. a, SpCas9 bound to 12-14 MM DNA highlighting the lack of REC3 residues contacting this region of the heteroduplex. REC3 is able to dock onto this mismatched DNA. b, FnCas9 scaRNA-mediated transcriptional repression structure with positions 12-14 mismatched. REC3 is unable to dock onto the truncated heteroduplex.

	FnCas9 PM Productive RuvC (EMD-48052) (PDB 9EHF)	FnCas9 PM Productive No RuvC (EMD-48053) (PDB 9EHG)	FnCas9 PM Non-Productive RuvC (EMD-48054) (PDB 9EHH)	FnCas9 PM Non-Productive No RuvC (EMD-48062) (PDB 9EHR)	FnCas9 16MM Productive (EMD-48070) (PDB 9EHX)	FnCas9 16MM Non-Productive (EMD-48069) (PDB 9EHW)	FnCas9 scaRNA gRNA 1101 DNA Non-productive (EMD-49074) (PDB 9N6T)
**Data collection and processing**							
Voltage (kV)	300 kV	300 kV	300 kV	300 kV	300 kV	300 kV	200 kV
Electron exposure (e–/Å^2)	80	80	80	80	80	80	50
Defocus range (μm)	-1.5 to -2.5	-1.5 to -2.5	-1.5 to -2.5	-1.5 to -2.5	-1.5 to -2.5	-1.5 to -2.5	-1.5 to -2.5
Pixel size (Å)	0.8332	0.8332	0.8332	0.8332	0.8332	0.8332	0.933
Symmetry imposed	C1	C1	C1	C1	C1	C1	C1
Initial particle images (no.)	1,956,741	1,956,741	1,956,741	1,956,741	2,495,949	2,495,949	1,573,500
Final particle images (no.)	277,939	48,457	51,749	94,982	79,858	63,288	283,943
Map resolution (Å)	2.6	3.0	2.9	2.9	2.9	3.0	2.9
FSC threshold	0.5	0.5	0.5	0.5	0.5	0.5	0.5
Map resolution range (Å)	2.6-6.0	3.0-6.7	2.9-6.7	2.9-6.7	2.9-6.4	2.9-6.4	2.8-6.2
**Refinement**							
Initial model used (PDB code)	5B2O	9EHF	9EHF	9EHG	9EHF	9EHH	AF3 model
Model resolution (Å)	2.8	3.0	3.1	3.1	3.1	3.2	2.9
FSC threshold	0.5	0.5	0.5	0.5	0.5	0.5	0.5
Map sharpening *B* factor (Å^2)	70.2	67.6	72.5	80.1	79.3	78.2	88.3
Model composition							
Non-hydrogen atoms	13,978	12,146	11,197	10,231	14,020	11,197	12,009
Protein residues	1,363	1,128	1,061	893	1,371	1,061	1,134
Nucleotides	133	133	117	133	132	117	125
Ligands	2 Mg^{2+}	2 Mg^{2+}	2 Mg^{2+}	2 Mg^{2+}	2 Mg^{2+}	2 Mg^{2+}	0
R.m.s. deviations							
Bond lengths (Å)	0.005	0.006	0.005	0.005	0.005	0.005	0.005
Bond angles (°)	0.911	1.139	0.981	0.987	1.019	1.030	0.970
Validation							
MolProbity score	0.93	1.10	1.00	1.05	1.05	0.92	0.83
Clashscore	1.72	3.11	2.26	2.68	2.67	1.68	1.16
Poor rotamers (%)	0.49	0.98	0.21	0.98	0.65	0.73	0.29
Ramachandran plot							
Favored (%)	98.44	98.38	98.09	98.64	98.16	98.66	98.04
Allowed (%)	1.56	1.62	1.91	1.36	1.84	1.34	1.96
Disallowed (%)	0.00	0.00	0.00	0.00	0.00	0.00	0.00

Supplementary Table S1: Cryo-EM data collection, refinement and validation statistics.

Name	Sequence (5’-3’)	Source
HBB_TS	/6-FAM/CATGGTGCATCTGACTCCTGAGGAGAAGTCTGCCGTTACTGCCCTGTGGGGCAAG	IDT
HBB_NTS	CTTGCCCCACAGGGCAGTAACGGCAGACTTCTCCTCAGGAGTCAGATGCACCATG	IDT
HBB_TS_1	/6-FAM/CATGGTGCATCTGACTCCTGAGGAGAAGTCTGCCGTTAGTGCCCTGTGGGGCAAG	IDT
HBB_NTS_1	CTTGCCCCACAGGGCACTAACGGCAGACTTCTCCTCAGGAGTCAGATGCACCATG	IDT
HBB_TS_2	/6-FAM/CATGGTGCATCTGACTCCTGAGGAGAAGTCTGCCGTTTCTGCCCTGTGGGGCAAG	IDT
HBB_NTS_2	CTTGCCCCACAGGGCAGAAACGGCAGACTTCTCCTCAGGAGTCAGATGCACCATG	IDT
HBB_TS_3	/6-FAM/CATGGTGCATCTGACTCCTGAGGAGAAGTCTGCCGTAACTGCCCTGTGGGGCAAG	IDT
HBB_NTS_3	CTTGCCCCACAGGGCAGTTACGGCAGACTTCTCCTCAGGAGTCAGATGCACCATG	IDT
HBB_TS_4	/6-FAM/CATGGTGCATCTGACTCCTGAGGAGAAGTCTGCCGATACTGCCCTGTGGGGCAAG	IDT
HBB_NTS_4	CTTGCCCCACAGGGCAGTATCGGCAGACTTCTCCTCAGGAGTCAGATGCACCATG	IDT
HBB_TS_5	/6-FAM/CATGGTGCATCTGACTCCTGAGGAGAAGTCTGCCCTTACTGCCCTGTGGGGCAAG	IDT
HBB_NTS_5	CTTGCCCCACAGGGCAGTAAGGGCAGACTTCTCCTCAGGAGTCAGATGCACCATG	IDT
HBB_TS_6	/6-FAM/CATGGTGCATCTGACTCCTGAGGAGAAGTCTGCGGTTACTGCCCTGTGGGGCAAG	IDT
HBB_NTS_6	CTTGCCCCACAGGGCAGTAACCGCAGACTTCTCCTCAGGAGTCAGATGCACCATG	IDT
HBB_TS_7	/6-FAM/CATGGTGCATCTGACTCCTGAGGAGAAGTCTGGCGTTACTGCCCTGTGGGGCAAG	IDT
HBB_NTS_7	CTTGCCCCACAGGGCAGTAACGCCAGACTTCTCCTCAGGAGTCAGATGCACCATG	IDT
HBB_TS_8	/6-FAM/CATGGTGCATCTGACTCCTGAGGAGAAGTCTCCCGTTACTGCCCTGTGGGGCAAG	IDT
HBB_NTS_8	CTTGCCCCACAGGGCAGTAACGGGAGACTTCTCCTCAGGAGTCAGATGCACCATG	IDT
HBB_TS_9	/6-FAM/CATGGTGCATCTGACTCCTGAGGAGAAGTCAGCCGTTACTGCCCTGTGGGGCAAG	IDT
HBB_NTS_9	CTTGCCCCACAGGGCAGTAACGGCTGACTTCTCCTCAGGAGTCAGATGCACCATG	IDT
HBB_TS_10	/6-FAM/CATGGTGCATCTGACTCCTGAGGAGAAGTGTGCCGTTACTGCCCTGTGGGGCAAG	IDT
HBB_NTS_10	CTTGCCCCACAGGGCAGTAACGGCACACTTCTCCTCAGGAGTCAGATGCACCATG	IDT
HBB_TS_11	/6-FAM/CATGGTGCATCTGACTCCTGAGGAGAAGACTGCCGTTACTGCCCTGTGGGGCAAG	IDT
HBB_NTS_11	CTTGCCCCACAGGGCAGTAACGGCAGTCTTCTCCTCAGGAGTCAGATGCACCATG	IDT
HBB_TS_12	/6-FAM/CATGGTGCATCTGACTCCTGAGGAGAACTCTGCCGTTACTGCCCTGTGGGGCAAG	IDT
HBB_NTS_12	CTTGCCCCACAGGGCAGTAACGGCAGAGTTCTCCTCAGGAGTCAGATGCACCATG	IDT
HBB_TS_13	/6-FAM/CATGGTGCATCTGACTCCTGAGGAGATGTCTGCCGTTACTGCCCTGTGGGGCAAG	IDT
HBB_NTS_13	CTTGCCCCACAGGGCAGTAACGGCAGACATCTCCTCAGGAGTCAGATGCACCATG	IDT
HBB_TS_14	/6-FAM/CATGGTGCATCTGACTCCTGAGGAGTAGTCTGCCGTTACTGCCCTGTGGGGCAAG	IDT
HBB_NTS_14	CTTGCCCCACAGGGCAGTAACGGCAGACTACTCCTCAGGAGTCAGATGCACCATG	IDT

HBB_TS_15	/6-FAM/CATGGTGCATCTGACTCCTGAGGACAAGTCTGCCGTTACTGCCCTGTGGGGCAAG	IDT
HBB_NTS_15	CTTGCCCCACAGGGCAGTAACGGCAGACTTGTCCTCAGGAGTCAGATGCACCATG	IDT
HBB_TS_16	/6-FAM/CATGGTGCATCTGACTCCTGAGGTGAAGTCTGCCGTTACTGCCCTGTGGGGCAAG	IDT
HBB_NTS_16	CTTGCCCCACAGGGCAGTAACGGCAGACTTCACCTCAGGAGTCAGATGCACCATG	IDT
HBB_TS_17	/6-FAM/CATGGTGCATCTGACTCCTGAGCAGAAGTCTGCCGTTACTGCCCTGTGGGGCAAG	IDT
HBB_NTS_17	CTTGCCCCACAGGGCAGTAACGGCAGACTTCTGCTCAGGAGTCAGATGCACCATG	IDT
HBB_TS_18	/6-FAM/CATGGTGCATCTGACTCCTGACGAGAAGTCTGCCGTTACTGCCCTGTGGGGCAAG	IDT
HBB_NTS_18	CTTGCCCCACAGGGCAGTAACGGCAGACTTCTCGTCAGGAGTCAGATGCACCATG	IDT
HBB_TS_19	/6-FAM/CATGGTGCATCTGACTCCTGTGGAGAAGTCTGCCGTTACTGCCCTGTGGGGCAAG	IDT
HBB_NTS_19	CTTGCCCCACAGGGCAGTAACGGCAGACTTCTCCACAGGAGTCAGATGCACCATG	IDT
HBB_TS_20	/6-FAM/CATGGTGCATCTGACTCCTCAGGAGAAGTCTGCCGTTACTGCCCTGTGGGGCAAG	IDT
HBB_NTS_20	CTTGCCCCACAGGGCAGTAACGGCAGACTTCTCCTGAGGAGTCAGATGCACCATG	IDT
HBB_TS_PT	/6-FAM/CATGGTGCATCTGACTCCTGAGGAGAAGTCTGCC*G*T*T*A*CTGCCCTGTGGGGCAAG	IDT
HBB_NTS_PT	CTTGCCCCACAGGGCAG*T*A*A*C*GGCAGACTTCTCCTCAGGAGTCAGATGCACCATG	IDT
1101_TS	ATTATCATCCTTATTTTTTATTATTATTAACATTGGATTTCTCCATTACAGCTAATTACTAAGTAAATAGC	IDT
1101_NTS	/6-FAM/GCTATTTACTTAGTAATTAGCTGTAATGGAGAAATCCAATGTTAATAATAATAAAAAATAAGGATGATAAT	IDT
scaRNA_gRNA	AGUAGGCGACGUAUAAUGUGGUUUCAGUUGCGCCGAAAGGCGCUCUGUAAUCAUUUAAAAGUAUUUUGAACGGACCUCUGUUUGACACGUCUG	IDT
HBB_gRNA	CAGUAACGGCAGACUUCUCCUCGUUUCAGUUGCGCCGAAAGGCGCUCUGUAAUCAUUUAAAAGUAUUUUGAACGGACCUCUGUUUGACACGUCUG	IDT

Supplementary Table S2: List of nucleotide sequences used in this study.

DNA substrate	k_{obs} (s^{-1})
PM	0.066
1 MM	0.074
2MM	0.045
3 MM	0.004
4 MM	0.007
5 MM	0.078
6 MM	0.149
7 MM	0.222
8 MM	0.102
9 MM	0.099
10 MM	0.086
11 MM	0.148
12 MM	0.027
13 MM	0.101
14 MM	0.012
15 MM	0.001
16 MM	0.036
17 MM	0.001
18 MM	0.034
19 MM	0.001
20 MM	0.001

Supplementary Table S3: FnCas9 observed cleavage rates for perfect match, and single mismatch (MM) DNA substrates.
